# Decoding surgical competence: surgical gesture analysis can determine competence in simulated VATS lobectomy, a validation study

**DOI:** 10.1007/s00464-026-13375-3

**Published:** 2026-10-01

**Authors:** Annarita Ghosh, Rikke Groth Olsen, Tamim Haidari, Lars Konge, Morten Bo Søndergaard Svendsen, Flemming Bjerrum, René Horsleben Petersen

**Affiliations:** 1 Department of Cardiothoracic Surgery, Rigshospitalet, Copenhagen University Hospital, Blegdamsvej 9, 2100 Copenhagen, Denmark; 2 Copenhagen Academy for Medical Education and Simulation (CAMES), the Capital Region of Denmark, Copenhagen, Denmark; 3 Copenhagen Prostate Cancer Center, Rigshospitalet, Copenhagen University Hospital, Copenhagen, Denmark; 4 Department of Clinical Medicine, University of Copenhagen, Copenhagen, Denmark; 5 Drug Delivery and Sensing (IDUN), DTU Health Tech, Technical University of Denmark, Kongens Lyngby, Denmark; 6 Gastrounit, Surgical Section, Copenhagen University Hospital - Amager and Hvidovre, Hvidovre, Denmark

**Keywords:** Surgical gestures, Simulation-based surgical assessment, Minimally invasive skill training, Objective skill assessment, Video-assisted thoracic surgeries, VATS

## Abstract

**Background:**

Minimally invasive thoracic surgery has become the standard of care, but is technically demanding, making objective assessment of technical competency essential during training. Video based *surgical gesture analysis* is a novel and objective method to evaluate technical skills during training for video-assisted thoracoscopic (VATS) lobectomies.

**Methods:**

VATS lobectomy videos performed on a virtual reality simulator were annotated using a predefined *surgical gesture* taxonomy including blunt dissection, sharp dissection, ribbon use, and stapler use. Gesture metrics included duration, frequency, idle time, and entropy. Principal component analysis and a support vector machine were used to generate gesture-based performance scores. Validity evidence was assessed against simulator-derived metrics and human expert ratings using the VATS assessment tool (VATSAT).

**Results:**

Surgical gesture analysis (SGA) was performed on 123 simulated VATS lobectomy procedures where 22 novices, 10 intermediates, and 9 experienced VATS surgeons performed three procedures each. Experienced surgeons had shorter procedure time (24.2 ± 9.2 min) than both intermediates (35.5 ± 9.9 min, *p* < 0.001) and novices (40.1 ± 14.3 min, *p* < 0.001), and mean gesture duration was significantly shorter for experienced (7 ± 1 s) compared with intermediates (8 ± 2 s, *p* = 0.026) and novices (11 ± 3 s, *p* < 0.001). Variability in gesture duration also decreased with experience (*p* < 0.001) and overall entropy showed significant group differences for novices compared with intermediates (*p* = 0.02) and experts (*p* = 0.049). Gestures correlated with simulator metrics and VATSAT scores, suggesting these reflected similar performance dimensions. SGA was able to classify experience level with a pass/fail threshold, achieving a sensitivity of 96.1% and specificity of 97.5%.

**Conclusion:**

SGA can be used to assess technical performance for simulated VATS lobectomies. It was able to differentiate between experience levels for multiple parameters, and has potential advantages compared with existing assessments such as rating scales and simulator metrics. Further studies are needed to explore the applicability on real procedures and the clinical implications of Surgical Gesture Analysis.

**Graphical abstract:**

**Figure:**
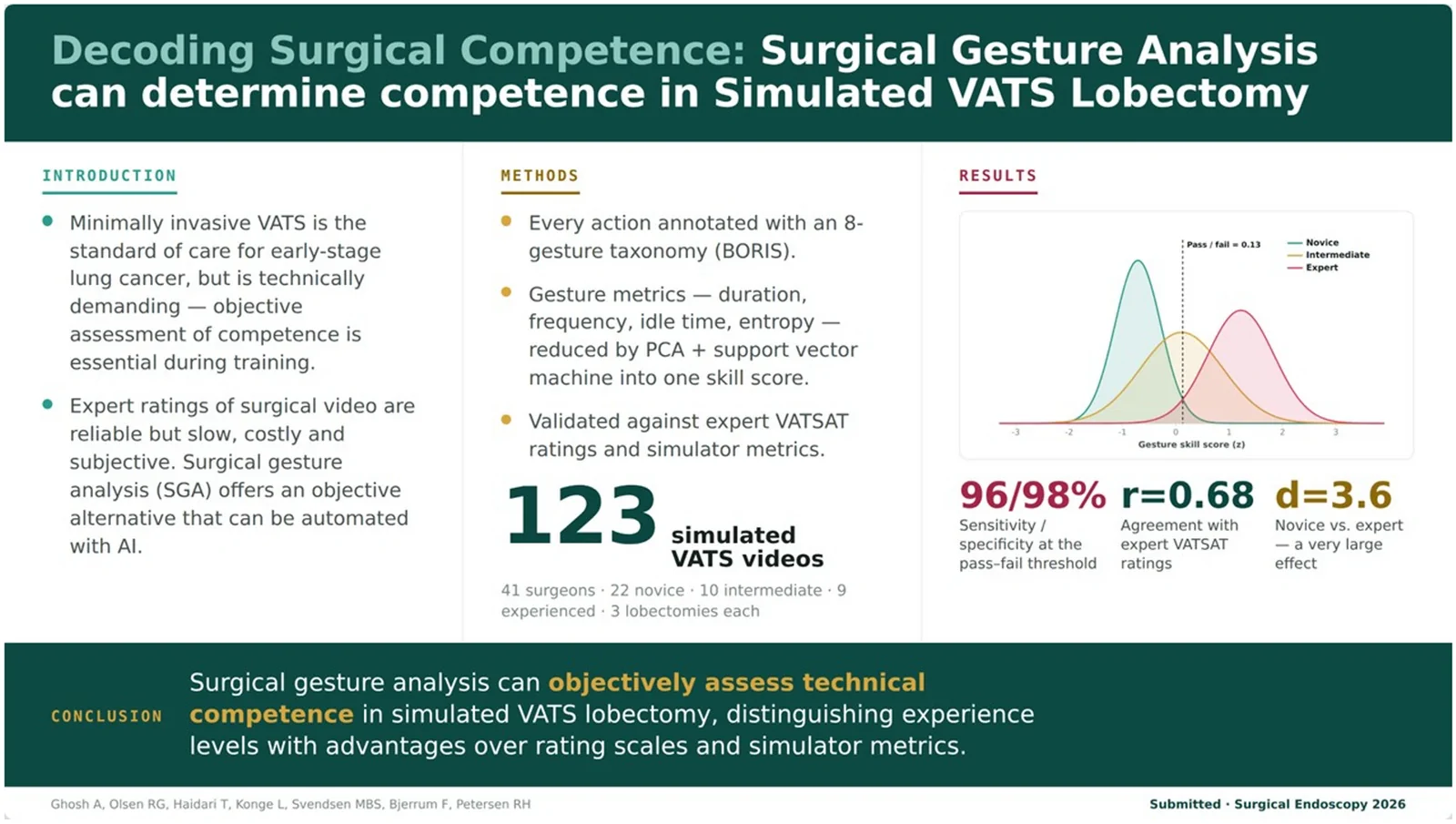

**Supplementary Information:**

The online version contains supplementary material available at https://doi.org/10.1007/s00464-026-13375-3.

Minimally invasive procedures, like video-assisted thoracoscopic surgery (VATS) and robotic-assisted thoracoscopic surgery (RATS) is the standard of care for the majority of early stage lung cancer [1, 2], but requires different psychomotor skills than open procedures. Surgeons’ performance directly influences patient outcomes and reliable and objective assessment methods during surgical training are essential to provide feedback and ensure competency [3–5].

In thoracic surgery, virtual reality (VR) simulation has become an important first step in surgical training by offering a safe and risk-free environment for surgical trainees to develop their skills [6, 7]. Simulators allow uninterrupted testing of trainees in totally standardized environments and provide performance parameters such as bleeding and economy of movement, which can be used to assess performance. However, these parameters measure cumulative efficiency and cannot capture how different parts of the procedure were performed.

Expert ratings of surgical videos using dedicated assessment tools such as the VATSAT (video-assisted thoracoscopic assessment tool) have proven to be a reliable assessment method for both simulated and real-life procedures [8]. Combining simulator performance parameters with experienced surgeons' ratings can provide a more encompassing evaluation of both the efficacy and quality of a procedure [9]. However, these approaches are time-consuming, costly, and susceptible to bias.

A more detailed and nuanced assessment form is needed, with the ability to give timely feedback and the potential to be used during supervised operations on patients.

One objective method of surgical skill assessment is video-based Surgical Gesture Analysis (SGA). The deconstruction of surgical procedures into smaller gestures, such as needle handling, tissue handling with blunt and sharp dissection and ensuring hemostasis, offers a nuanced approach to performance assessment and can potentially be used to provide live feedback during training using artificial intelligence to perform the analysis [10–14]. This can remove the time-consuming and costly reliance on expert ratings and give objective insights into technical performance; however, the method needs to be tested before using it as an assessment tool.

In this study, we examined validity evidence for the assessment of simulated thoracoscopic surgeries using surgical gestures analysis (SGA). We investigated whether gesture-based metrics accurately reflect technical competence and can be meaningfully interpreted for use in surgical training and assessment.

## Materials and methods

### Study design

We evaluated the validity evidence for using Surgical Gesture Analysis using Messick’s unitary framework (content, response process, internal structure, relationship with other variables, and consequences) [15].

### Ethics

The study was deemed exempt from ethical approval by the Committee on Health Research Ethics for the Capital Region of Denmark (journal no.: H-2405F906).

### Data material

We used 123 video recordings of simulated lobectomies performed on the VATS module on the Lapsim® virtual reality simulator (Surgical Science, Gothenburg, Sweden) from a previously published study [6]. The data collection and data analysis were likewise described using Messick’s framework.

The videos included simulated lobectomies of all five lung lobes using the standardized anterior Copenhagen surgical approach [1]. Surgeries were performed by 41 participants (22 novices, 10 intermediates, and 9 experienced VATS surgeons) who completed lobectomies of three random, different lobes. The cohort was defined from the previous studies of VATSAT on simulated lobectomies [6, 7, 9]. *Novices* were medical students with no surgical experience, *intermediates* were surgical trainees, who had performed less than 100 minor VATS procedures (e.g., wedge resection, pleural biopsy) and 0–50 VATS lobectomies, with a mean of 5 lobectomies, and *experienced* VATS surgeons who had done more than 50 VATS lobectomies.

All videos had been rated with a modified version of the VATSAT (Table 1), with three items excluded since the simulator can’t simulate tumor extent, nor lymph and lobe removal [16]. The ratings were done by three experienced VATS surgeons blinded to the participants’ identities, resulting in 369 ratings, with a pass/fail level of 19 points [6–9].

**Table 1:** Modified VATSAT [8] and Simulator metrics [6]

Modified VATSAT	Simulator metrics
• Dissection of hilum and veins • Dissection of arteries • Dissection of bronchus • Respect for tissue and structure • Technical skills in general	• Total procedure time (s) • Blood loss (ml) • Total instrument path length (m)

The modified VATSAT consists of five items which are scored from 1 to 5. The simulator metrics consist of 3 automatically recorded metrics, which were then combined with the expert rating for a composite score

### Surgical gesture taxonomy and annotation (content and response process)

For video analysis, we developed a taxonomy for gesture annotation of VATS lobectomies. Gestures were defined as distinct, recognizable actions used to deconstruct and classify surgical performance for detailed analysis and skill assessment [17]. As no taxonomy has been defined for VATS yet, the gesture types used in the simulated VATS videos were agreed upon by the investigator group. The taxonomy consisted of eight gestures (Table 2). Each surgical movement in the surgical videos was annotated accordingly to ensure detailed and precise logging. Time not spent on a gesture was labeled as “idle time”.

**Table 2:** Surgical gesture descriptions

Blunt dissection with whiskers, Baltimore, suction, etc. pushing and dissecting the tissue
Sharp dissection with scissors, hook, Maryland to cut, burn and dissect the tissue
Presentation with ribbon around the anatomical structure such as vein, artery, or bronchus
Removal of ribbon before stapling
Placement of stapler around the anatomical structure such as vein, artery or bronchus
Presentation of stapler end to ensure correct placement
Closing of stapler with the structure in the stapler line
Application of stapler with cutting and release of the structure
*Idle time: Time not spent doing any of the above gestures

^*^Only used for statistical analysis, not included in heat map or histogram

**Fig. 1:**
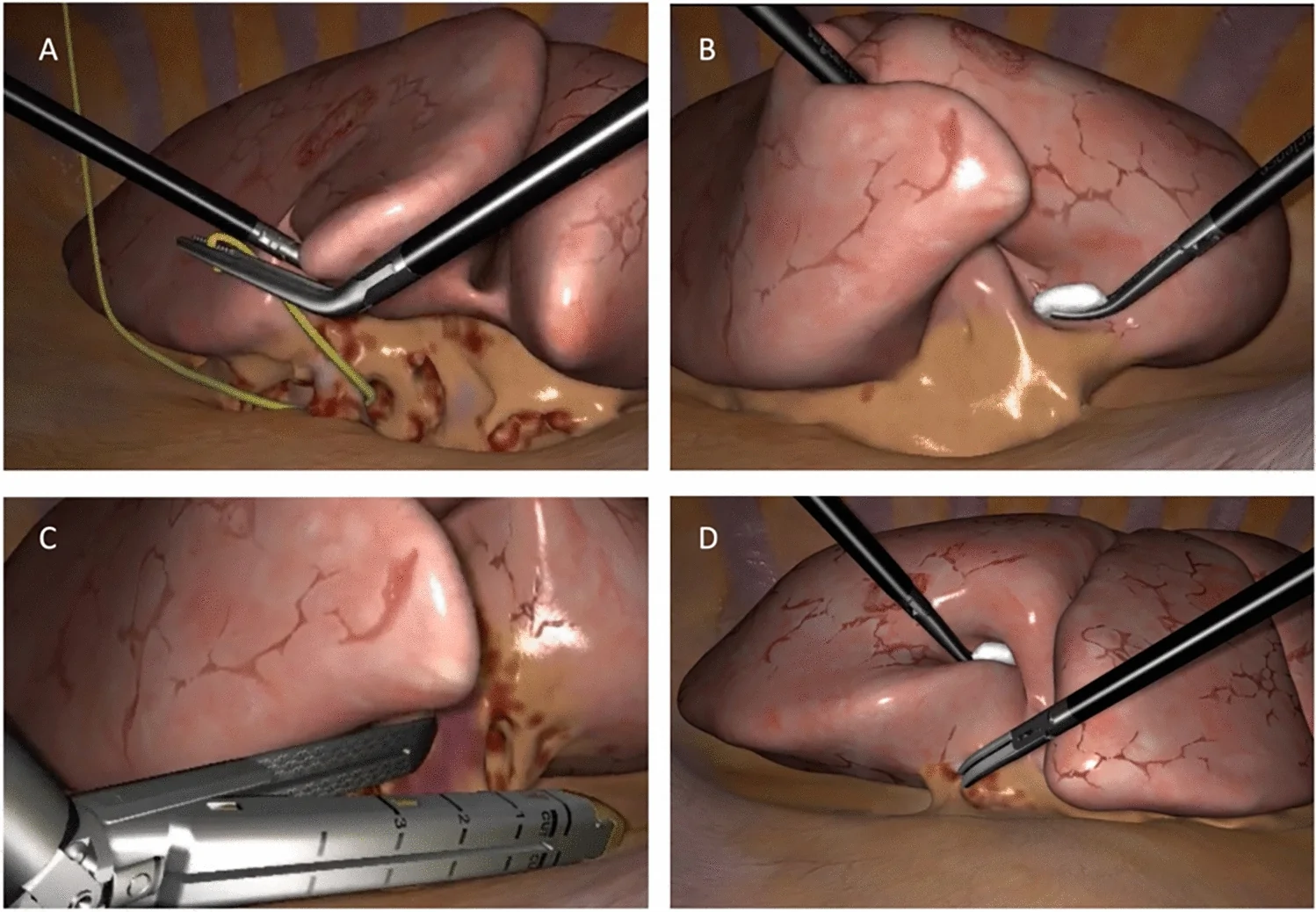
Examples of four different gestures, screenshots from the simulated lobectomy videos. **A** Presentation of ribbon, **B** blunt dissection with whisker, **C** placement of stapler, **D** sharp dissection using curved Maryland dissector

All videos were annotated by one investigator (AG) using BORIS: Behavioral Observation Research Interactive System v. 7.12.2 [18], an event-logging software for video coding. To ensure correct annotation, a sample set of annotated gestures was reviewed and validated by one expert surgeon (RHP) who had performed more than 2000 VATS lobectomies. Each gesture was time-stamped with start and stop times. The beginning of each gesture was annotated when the instrument moved toward the structure and ended as the instrument moved away. Visual examples of gestures can be found in Fig. 1 and Supplementary Video 1.

### Statistical analysis

All analyses and visualizations were completed using the Python programming language (version 3.10.10, Python Software Foundation, Amsterdam, Netherlands, www.python.org).

### Descriptive and entropy-based gesture patterns

From the annotated videos, procedure time was extracted, and idle time was calculated as the time between each gesture. Frequency of gestures was counted as the onset of each gesture event for the number of total gestures and at the onset of each gesture type. Differences in all descriptive measures across experience levels were assessed using Welch’s one-way analysis of variance (ANOVA) to account for unequal variances, followed by pair-wise Welch’s t-tests.

Overall gesture entropy was measured with Shannon Entropy, a unitless measurement quantifying the overall variability of gesture frequency, getting gesture probabilities by counting the frequency of each gesture and normalizing by the total count, then the overall uncertainty was quantified using the Shannon formula [19]. A high entropy count would indicate a high variation of gestures, while a lower entropy score would indicate a more focused and structured use of gestures (see Table 3).

Markov Chain Transition Entropy was used to evaluate the sequence of gesture transitions, as it reflects how predictable or random the sequence of gesture transitions was, with a higher number reflecting a higher uncertainty [20].

### Surgical gesture analysis (internal structure)

First, all procedural annotations were interpolated to a fixed length, obtaining a normalization (percent) of observations from procedural length.

Gesture patterns were visualized on heat maps, “*snail tracks*”, with the distribution of gestures illuminated and idle time dark on the normalized fixed-length bars [11].

A Principal Component Analysis (PCA) was applied to normalized gesture data to reduce dimensionality while retaining variance. A support vector machine (SVM) was trained using the PCA-transformed data along with the expertise labels on experienced VATS surgeons. This allowed us to calculate an expertise probability for each participant. The mean and standard deviation for the probabilities were calculated for all participants and used to calculate z-scores, representing each participant’s performance as the deviation from the average VATS performance.

### Performance assessment (consequences)

To calculate a pass/fail score for assessing surgical competence using gestures, we used the contrasting groups’ standard setting method by generating normal distributions of z-scores for novices and experienced [21]. Using the properties of the normal distribution, the probabilities of experienced scoring above the threshold (true positives) and novices scoring below the threshold (true negatives) were estimated. From these probabilities, sensitivity, specificity, positive predictive value (PPV), and negative predictive value (NPV) were calculated under the assumption of equal prevalence of novices and experienced VATS surgeons. A theoretical confusion matrix was constructed based on samples of 100 novices and 100 experienced VATS surgeons.

We investigated the model’s ability to detect the different levels of experience between the three groups by using Welch’s t-tests and Cohen’s effect sizes for pairwise comparisons, novice vs. intermediates, intermediates vs. experienced VATS surgeons, and novices vs. experienced VATS surgeons.

### Comparison with established assessment methods (relationship to other variables)

To standardize and compare the gesture results with VATSAT and the simulator scores, z-scores were calculated for all three different datasets. For VATSAT and simulator metrics, the dataset was normalized using the expert group's mean and standard deviation.

To investigate the relationship between the three assessment methods, we used linear regression analyses and Bland–Altman plots [22] to assess agreement. This method allowed us to assess the level of agreement and identify any systematic bias between the measurement approaches.

## Results

### Gesture patterns across experience levels

Experienced VATS surgeons were significantly faster in completing the procedure (mean 24.2 ± 9.2 min) than both novices (mean 40.1 ± 14.3 min, *p* < 0.001) and intermediates (mean 35.5 ± 9.9 min, *p* < 0.001), but there was no significant difference between novices and intermediates (*p* = 0.12).

The overall ANOVA did not show a statistically significant different idle time between groups (p = 0.098), but a pairwise comparison indicate higher idle time in novices compared to experienced VATS surgeons (1279 ± 410 vs. 775 ± 307 s, *p* = 0.03).

The gesture frequency (counts) was significantly higher for intermediates (144 ± 52) compared to both novices (117 ± 37, p = 0.039) and experienced VATS surgeons (112 ± 41, *p* = 0.031), while there was no significant difference between novices and experienced VATS surgeons (*p* = 0.68).

Experienced VATS surgeons had a significantly shorter mean gesture duration (7 ± 1 s) than both novices (11 ± 3 s, *p* < 0.001) and intermediates (8 ± 2 s, *p* = 0.026), with intermediates also having a significant shorter mean duration than novices (*p* < 0.001). A similar pattern was found for the variability of gesture durations, with experienced VATS surgeons showing significantly less variability (8 ± 2 s) than both intermediates (12 ± 4 s, *p* = 0.003) and novices (17 ± 7 s, *p* < 0.001), and intermediates differing significantly from novices (*p* < 0.001).

**Fig. 2:**
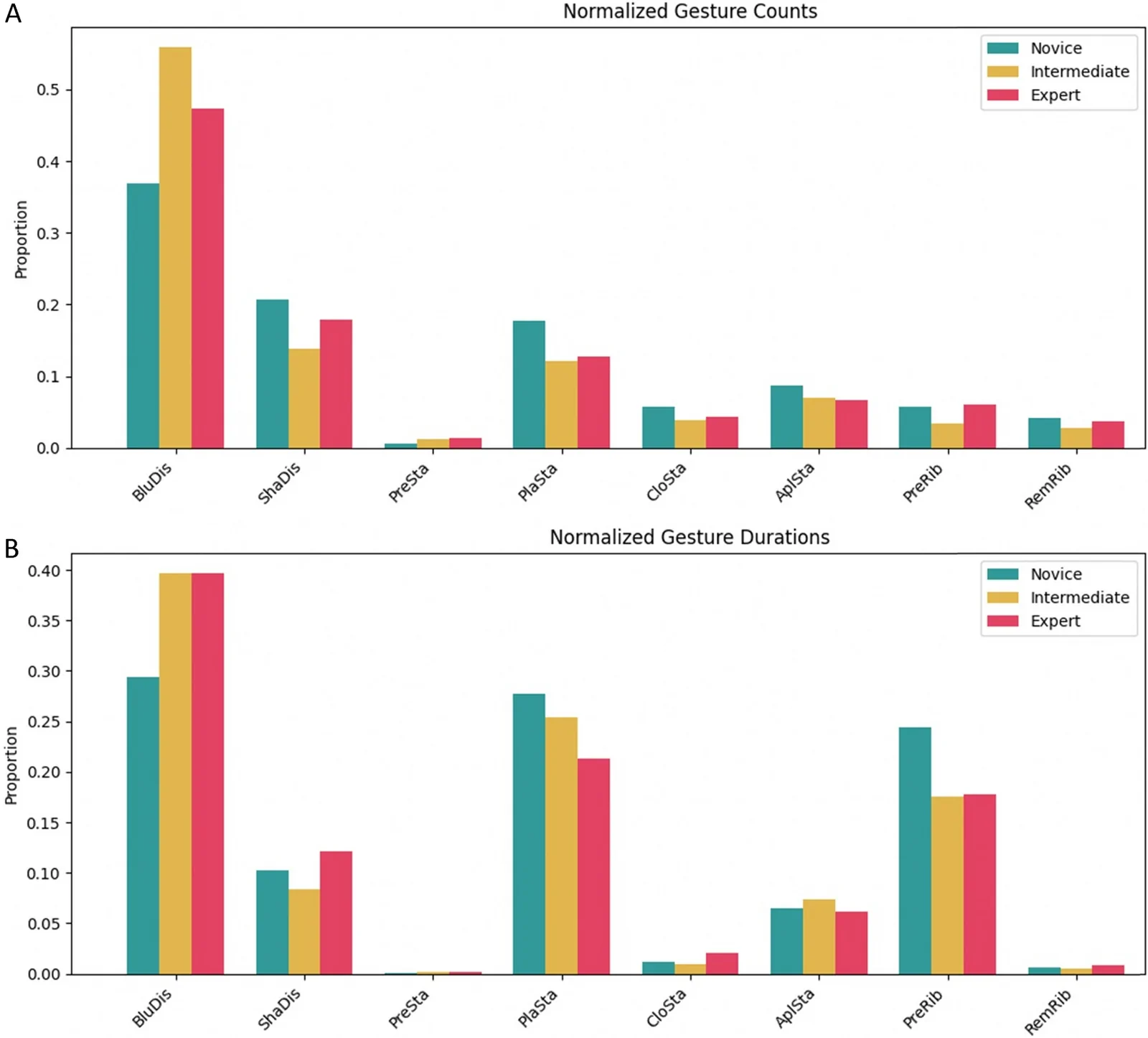
**A**, **B** Histogram of the proportion of counts and durations of the eight gestures divided into subgroups of novice, intermediate and experienced VATS surgeons (experts). *BluDis* blunt dissection, *ShaDis* sharp dissection, *PreSta* preparation of stapler, *PlaSta* placement of stapler, *CloSta* closing of stapler, *AplSta* application of stapler, *PreRib* preparation of ribbon, *RemRib* removal of ribbon

Of the eight different gesture types, the most used for novices, intermediates, and experienced VATS surgeons were blunt dissections, both in duration and frequency (Fig. 2). Intermediates used blunt dissection the most, followed by experienced VATS surgeons and novices.

**Fig. 3:**
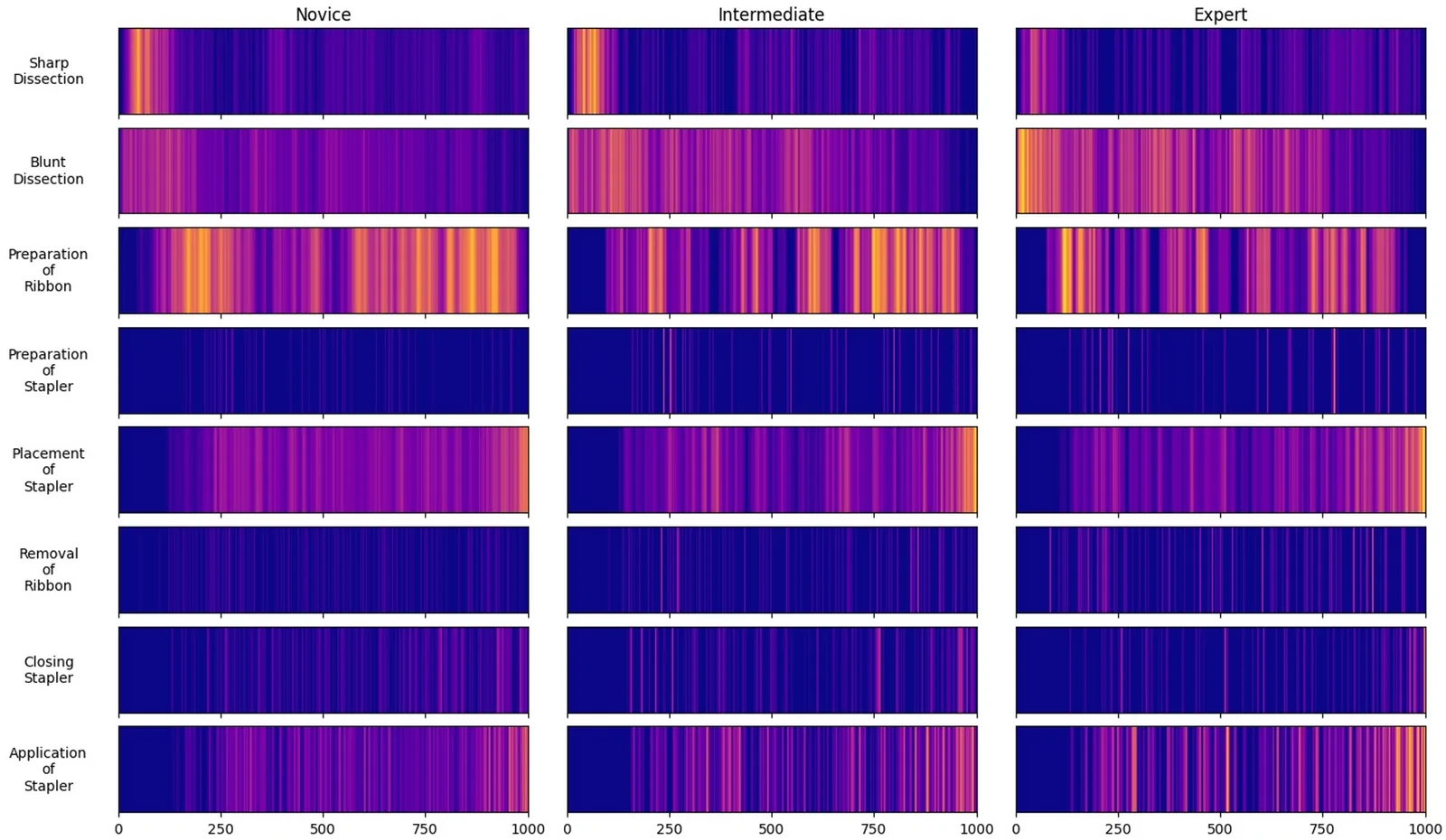
Heat maps, or snail tracks, for the mean distributions of gestures chronologically throughout the simulated lobectomies across experience levels, with all procedural annotations interpolated to a fixed length, obtaining a normalization (percent) of observations. Each vertical line represents the active time of the corresponding gesture. The brighter, the more activity. Solid blue means the gesture is not in use

Novices took significantly longer in preparing the ribbon before placing the stapler (*p* < 0.001) but did not use the gesture more than experts (*p* = 0.90). Proportionally, novices used preparation of stapler significantly less both in duration (*p* = 0.04) and frequency (*p* = 0.005) than experienced VATS surgeons, while using more time (*p* < 0.001) and counts (*p* = 0.012) in the placement of the stapler and the same was seen in the application of staplers with novices using more counts (*p* = 0.02) and duration (*p* < 0.001). The distribution of gestures is visualized as a one-dimensional heat map for the three groups (Fig. 3).

The average time between different gesture types, from the beginning of one gesture type, such as sharp dissection, to another gesture type such as blunt dissection, varied significantly across groups (ANOVA *p* < 0.001); experts (166 ± 70 s) exhibited a significantly higher mean than novices (107 ± 34 s, *p* < 0.001) and intermediates (119 ± 40 s, *p* = 0.008), whereas the difference between novices and intermediates did not reach significance (*p* = 0.24).

### Gesture entropy

Novices had a more scattered use of gestures measured with Shannon entropy (2.33 ± 0.28) compared to intermediates (2.15 ± 0.29, *p* = 0.02) and experienced VATS surgeons (2.21 ± 0.22, *p* = 0.049) who showed a more focused use of specific gestures. Higher entropy reflects how gestures are distributed but does not imply better performance. The Markov Chain Transition Entropy, which reflects how predictable or random the sequence of gesture transitions is, did not exhibit any significant group differences (ANOVA *p* = 0.37), and none of the pairwise comparisons were significant (see Table 3).

**Table 3:** Descriptive procedure and gesture characteristics

	Experience group			ANOVA	Pairwise comparison (*t*-test)		
	Novice (*n* = 22)	Intermediate (*n* = 10)	Experienced (*n* = 9)		N vs I	N vs E	I vs E
Total procedure time (min)	40.1 ± 14.3	35.5 ± 9.9	24.2 ± 9.2	**< 0.0001**	0.12	**< 0.001**	**0.0003**
Total idle time (min)	21.3 ± 6.8	18.9 ± 6	12.9 ± 5,1	0.098			
Time between gesture types (s)	107 ± 34	119 ± 40	166 ± 70	**< 0.001**	0.24	**0.0005**	**0.0081**
Gesture frequency (counts)	117 ± 37	144 ± 52	112 ± 41	**0.025**	**0.039**	0.68	**0.031**
Gesture duration (s)	11 ± 3	8 ± 2	7 ± 1	**< 0.001**	**< 0.0001**	**< 0.0001**	**0.026**
Overall duration variability (s)	17.40 ± 7.33	11.79 ± 4.40	8.32 ± 2.28	**< 0.001**	**0.0003**	**< 0.0001**	**0.0028**
Gesture entropy (Shannon, unitless)	2.33 ± 0.28	2.15 ± 0.29	2.21 ± 0.22	**0.024**	**0.02**	**0.049**	0.43

Comparing novice, intermediate and experienced VATS surgeons’ means ± SD. Pairwise comparisons where ANOVA indicated statistically significant group differences. Numbers are highlighted in bold when the *p*-value is significant (*p* ≤ 0.05). *N* novices, *I* intermediates, *E* experienced

### Gesture-based performance score and pass/fail limit

**Fig. 4:**
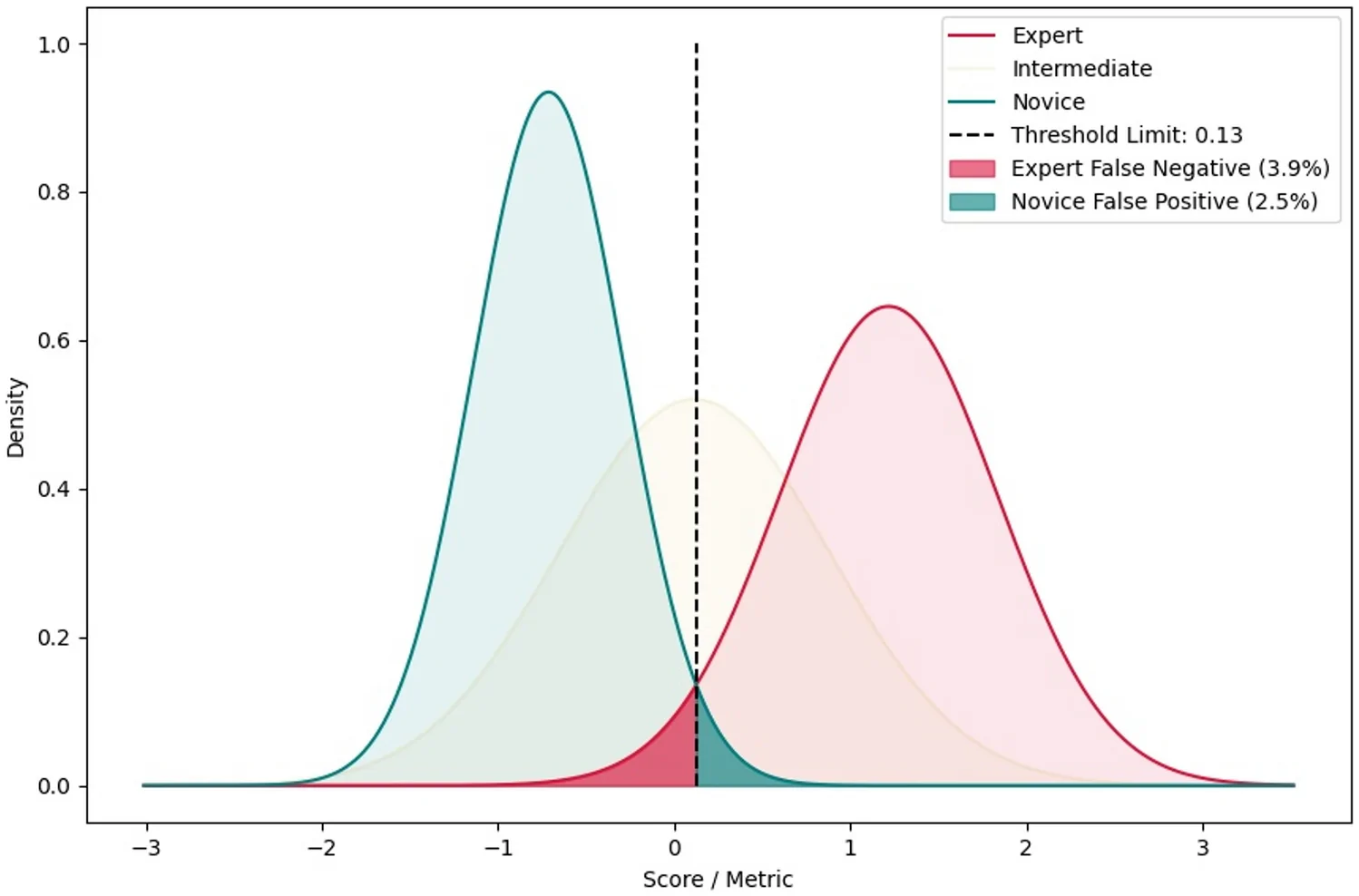
Pass/fail limit with bell curves for novices, intermediates, and experienced VATS surgeons showing a pass/fail limit of 0.13 with 96.1% of experienced VATS surgeons passing the test and 97.5% of the novices failing

Normalized gesture data for each procedure (Idle time, Total procedure duration, Mean interval between gesture events, SD of intervals between gesture events, Total number/frequency of gesture events, Mean gesture duration, SD of gesture duration, Number of gesture onsets/shifts) were reduced to principal components and converted into summary z-scores using the PCA/SVM-based gesture model. The distributions of summary z-scores were as follows: experienced VATS surgeons had a mean of 1.22 ± 0.62, intermediates had a mean of 0.11 ± 0.77, and novices had a mean of − 0.71 ± 0.43. The contrasting groups’ standard setting method established a pass/fail limit of 0.13 (Fig. 4).

The gesture-based assessment has an estimated sensitivity of 96.1% and a specificity of 97.5%.

Using Cohen’s d to calculate effect sizes between the three groups, we found that there was a very large effect between novices and experienced VATS surgeons (*d* = 3.6), and a large effect between both novices and intermediates (*d* = 1.3) and intermediates and experienced VATS surgeons (*d* = 1.6), meaning that the differences between groups were not only statistically significant but also meaningful, with clear separation in assessed performance levels.

### Correlation with other assessment tools (VATSAT and simulator metrics)

**Fig. 5:**
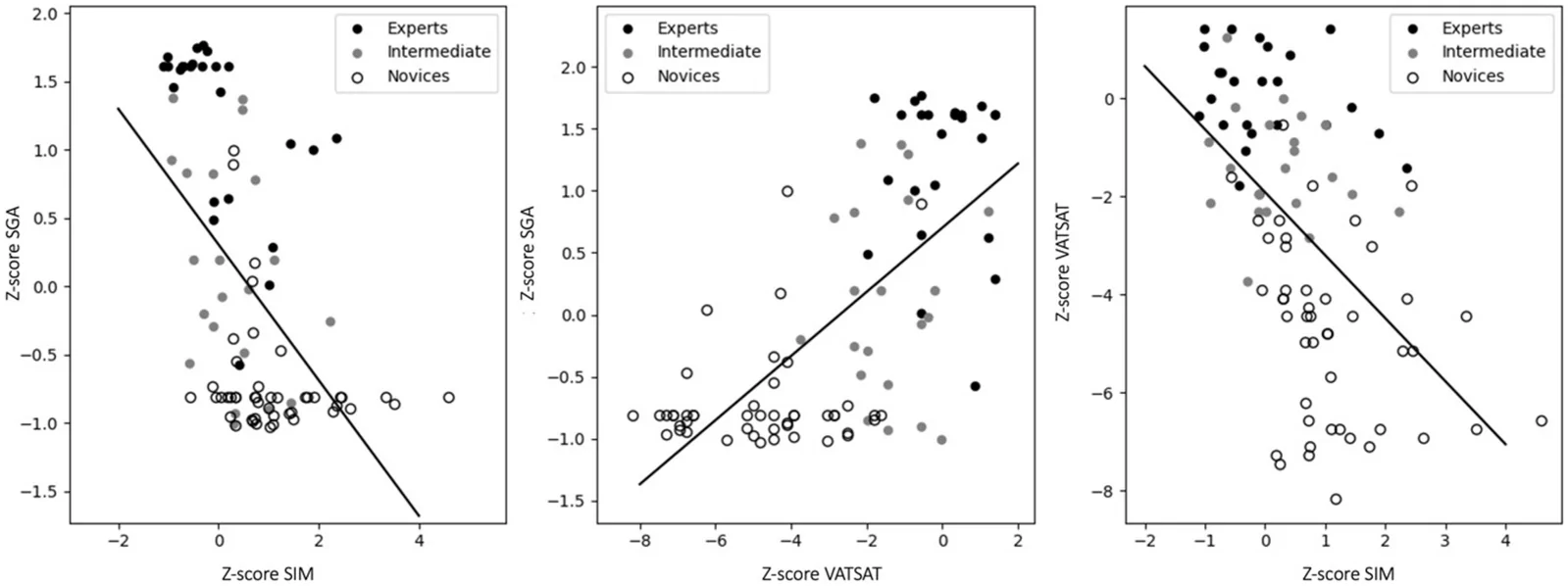
Correlation of Z-scores comparing SGA, simulator metrics, and VATSAT. *SIM* simulator metrics, *VATSAT* VATS lobectomy assessment tool, *SGA* surgical gesture analysis

When comparing simulator metrics with gesture analyses using the linear regression analysis, we determined a significant negative correlation (slope = − 0.50, *r* = − 0.54, *p* = < 0.001) with a modest fixed bias (mean difference = 0.62 ± 1.81) indicating that gesture scores increased when simulator performance parameters decreased, reflecting that lower simulator metrics indicate a higher proficiency level (less bleeding, faster performance, and better economy of movement). When comparing VATSAT scores with gestures, we found a positive correlation between the two methods (slope = 0.26, *r* = 0.68, *p* = < 0.001). The bias statistics showed a mean difference of − 2.71 ± 2.06 (Fig. 5).

## Discussion

This study provides validity evidence for Surgical Gesture Analysis (SGA) as an assessment method for VATS lobectomies that can distinguish between novices, intermediates, and experienced surgeons with a reliable pass/fail classification. Notably, SGA offers something beyond traditional simulator metrics: it provides a structured, objective way of breaking down procedures into smaller, interpretable elements.

The surgical gesture patterns suggest that increased experience was reflected in a more structured and efficient use of surgical gestures, with a progressive understanding of the surgical steps. Novices changed gesture types more often, spent more time on each gesture as well as on the total procedure, and had a more varied use of gestures, even though the overall sequence of steps remained stable across groups. As an example, a novice will also use the ribbon before stapling, but might have more gestures placing the ribbon, and might have to go back to dissecting some more before correctly placing the ribbon, have trouble placing the stapler at the right angle, and thus start over in placing the ribbon from another angle, while an experienced surgeon have a better understanding of the angles, and can thus more directly go from placing the ribbon to placement of the stapler.

Experienced surgeons used more time on one gesture type before switching to a different gesture, while still being overall faster, reflecting fewer instrument switches and a more efficient use of each gesture (“finish what you started before moving to the next step”).

This is exemplified in that the experienced surgeons used more time preparing the stapler but fewer takes and time on both the placement and the application of the stapler, highlighting that they were better able to think ahead, prepare, and thus more effectively use the stapler. This mirrors findings from robot-assisted surgery, where increasing experience was similarly associated with fewer but more purposeful gestures [11, 23].

SGA showed meaningful insights for intermediates as well. Intermediates used blunt dissection most frequently, reflecting a stage in training where they rely on careful blunt tissue manipulation to avoid injury, progressing slowly through the procedure. As a proportion of total procedure time, experienced surgeons used a comparable share of time on blunt dissection as intermediates. Combined with using fewer counts, experienced surgeons stayed in the same gesture type for a longer time, using it more effectively.

While intermediates showed mixed use of gesture types and patterns in gesture use, some resembling experts, others resembling novices, this likely reflects the transitional nature of this group and illustrates that gesture-based metrics are sensitive to subtle differences in performance.

This aligns with established research from other surgical specialties, confirming that gesture patterns reflect surgical skill [14, 24, 25]. Ma et al. furthermore demonstrated that surgical gestures could serve as a predictor of patient outcomes for clinical urological procedures [25].

The gesture-based scores correlated positively with VATSAT and inversely with simulator metrics, where lower simulator scores indicate higher proficiency. Olsen et al., who found moderate agreement (ICC = 0.70) between automated performance metrics and surgical gestures in simulated robot-assisted radical prostectomy [23] and Hung et al. who found low correlations between kinematic performance metrics and the expert global rating tool GEARS [10]. This suggests gesture analysis raises the question of whether gesture analysis better reflects expert ratings than purely kinematic metrics.

In this study, gestures were manually annotated, but with Artificial Intelligence (AI) the time-consuming gesture annotations can potentially be done automatically and used real-time during training as well as possibly for intraoperative guidance and assessment [12, 13, 26]. AI has already shown that it can extract information on surgical skills from unannotated videos, providing immediate feedback to surgeons and establishing a link between intraoperative and postoperative outcomes [27]. In thoracic surgery, AI has been shown useful in identifying anatomical structures as well as classifying procedural phases in video analysis [28]. For clinicians, this means that skills assessment can move from being subjective and delayed (expert scorings after reviewing full videos) to becoming automated, objective, immediate and relevant to the surgical steps in the future [23].

Taken together, these findings highlight SGA as a potentially powerful tool for surgical education and assessment. SGA can address two central challenges in VATS training; formative assessment during training and summative assessment at the end of training in the form of automatic and objective assessment and certification. This could support a mastery learning framework were trainees progress based on demonstrated competence rather than at a fixed time or after a certain number of cases [29].

This makes it a strong candidate for integration into future training programs, where timely and objective assessment is critical for both surgeon development and patient safety [30].

### Strengths

To our knowledge this is the first study that investigates SGA for assessing competence in VATS lobectomies. We used Messick’s framework to ensure a standardized reporting of a novel assessment.

We have performed detailed gesture annotation and triangulation with both expert ratings and simulator metrics. The inclusion of participants across three experience levels enabled robust group comparisons, and the use of PCA, SVM, and z-scores supports transparency and interpretability of the classification model.

All videos were blinded before assessment, both in the dataset with VATSAT from three experienced raters, and in our study before annotation. Even though gesture annotations were performed by a single trained rater (AG), videos of gestures were cross-validated by an expert surgeon (RHP) to ensure the response process. Our study was done using simulated videos, which enabled a standardized environment with a random sequence of lobes for all participants, while ensuring that novices could be included and demonstrate their real level of surgical competency without concerns for patient safety.

### Limitations

We were not able to cross-validate all videos with gestures annotations from either another expert surgeon or an independent rater and calculate interrater agreement, thereby risking annotator-bias. However, this is very common in gesture analysis due to the time-consuming nature of manual annotations.

This study was a continuation of previous studies with the strength and limitations of the existing dataset. Novices were medical students with no prior surgical experience, as such a difference in skill between experts and novices would be expected. This expected difference in known-groups is part of the validity evidence, the relationship to other variables, and well-known and -used in the literature [31, 32]. However, on its own the two groups are very different from each other, and differ in many other constructs as well, therefore the usefulness of novice-expert comparisons has been debated [33]. Our study also investigated intermediates, a group with some surgical knowledge, who hadn’t mastered VATS lobectomies yet, and found significant differences in the intermediate-expert comparisons as well.

The present study could have a concern in the relatively small sample of experienced surgeons, as highly specialized thoracic surgeons are scarce and difficult to recruit, also evident in that similar assessment and annotation studies typically rely on comparable sample sizes, for comparison Olsen et al. used 9 and Ma et al. 21 experienced [11, 25]. As it was done on simulated videos it is not certain that results from this study can be translated to real-world surgeries.

### Future perspectives

SGA as an assessment method which is quickly evolving and expanding across different surgical specialties [24]. Studies have shown that gestures can be used to predict patient outcomes in robotic-assisted prostatectomies [14, 25]. While saving time in the assessment method, the interpretation of the gesture analysis could potentially be harnessed for feedback both in simulated environments during training, but also in real-world surgeries, opening the possibility for real-time feedback and guidance.

Thus, SGA can potentially be used in the clinical setting as well as simulated environment, tracking progressive skills acquisition throughout clinical training of thoracic surgeons. It would therefore be interesting to investigate gesture assessment on real-life video-assisted thoracoscopic surgeries.

## Conclusion

Gesture-based assessment in simulated VATS lobectomies could differentiate experience level on multiple parameters and provided more descriptive insights into procedural performance compared to simulator metrics. SGA provide detailed insights into the technical aspects of surgery and has the potential to be used for assessment and automated, detailed interpretation of surgical skill.

## Supplementary Information

Below is the link to the electronic supplementary material.

Supplementary file1 (MP4 98841 KB)
